# Human Amnion Epithelial Cells and Their Derived Exosomes Alleviate Sepsis-Associated Acute Kidney Injury *via* Mitigating Endothelial Dysfunction

**DOI:** 10.3389/fmed.2022.829606

**Published:** 2022-03-24

**Authors:** Dongxuan Chi, Ying Chen, Chengang Xiang, Weijian Yao, Hui Wang, Xizi Zheng, Damin Xu, Nan Li, Min Xie, Suxia Wang, Gang Liu, Shuangling Li, Li Yang

**Affiliations:** ^1^Department of Critical Care Medicine, Peking University First Hospital, Beijing, China; ^2^Renal Division, Peking University First Hospital, Beijing, China; ^3^Institute of Nephrology, Peking University, Beijing, China; ^4^Key Laboratory of Renal Disease, Ministry of Health of China, Beijing, China; ^5^Key Laboratory of CKD Prevention and Treatment (Peking University), Ministry of Education of China, Beijing, China; ^6^Research Units of Diagnosis and Treatment of Immune-Mediated Kidney Diseases, Chinese Academy of Medical Sciences, Beijing, China; ^7^Laboratory of Electron Microscopy, Pathological Center, Peking University First Hospital, Beijing, China

**Keywords:** hAECs, exosomes, sepsis, acute kidney injury, endothelial dysfunction, stem cell therapy

## Abstract

**Background:**

Sepsis is characterized by organ dysfunction resulting from a patient’s dysregulated response to infection. Sepsis-associated acute kidney injury (S-AKI) is the most frequent complication contributing to the morbidity and mortality of sepsis. The prevention and treatment of S-AKI remains a significant challenge worldwide. In the recent years, human amnion epithelial cells (hAECs) have drawn much attention in regenerative medicine, yet the therapeutic efficiency of hAECs in S-AKI has not been evaluated.

**Methods:**

Septic mice were induced by cecal ligation and puncture (CLP) operation. hAECs and their derived exosomes (EXOs) were injected into the mice *via* tail vein right after CLP surgery. The 7-day survival rate was observed. Serum creatinine level was measured and H&E staining of tissue sections were performed 16 h after CLP. Transmission electron microscopy was used to examine the renal endothelial integrity in CLP mice. Human umbilical vein endothelial cells (HUVECs) were treated with lipopolysaccharide (LPS) and EXOs. Zonula occludens-1 (ZO-1) localization was observed by immunofluorescence staining. Expression of phosphor-p65 (p-p65), p65, vascular cell adhesion molecule-1 (VCAM-1), and ZO-1 in the kidney were determined by Western blot.

**Results:**

hAECs decreased the mortality of CLP mice, ameliorated septic injury in the kidney, and improved kidney function. More precisely, hAECs suppressed systemic inflammation and maintained the renal endothelial integrity in septic animals. EXOs from hAECs exhibited similar renal protective effects as their parental cells. EXOs maintained endothelial cell adhesion junction *in vitro* and inhibited endothelial cell hyperactivation *in vivo*. Mechanistically, EXOs suppressed proinflammatory nuclear factor kappa B (NF-κB) pathway activation in LPS-treated HUVECs and in CLP mice kidneys.

**Conclusion:**

Our results indicate that hAECs and their derived EXOs may ameliorate S-AKI *via* the prevention of endothelial dysfunction in the early stage of sepsis in mice. Stem cell or exosome-based therapy targeting endothelial disorders may be a promising alternative for treatment of S-AKI.

## Introduction

Sepsis is defined as life-threatening organ dysfunction caused by a dysregulated host response to infection (1) and remains a major cause of health loss worldwide. Sepsis-associated acute kidney injury (S-AKI) is a frequent complication and contributes to the high morbidity and mortality of the critically ill patients (2). Angus et al. examined 192,980 patients with severe sepsis from seven US states and found that AKI occurred in 22% and was associated with a mortality of 38.2% (3). A Chinese retrospective study of 146,148 patients defined AKI in 47.1% of septic cases (4). Even patients survive from sepsis, the lifetime risk of chronic kidney disease (CKD) and end-stage kidney disease is higher.

The pathogenesis of S-AKI is complex and has unique features (2). Observations in humans and animals with sepsis indicate three main alterations in organs: the microcirculatory flow abnormalities, the dysregulated inflammatory response, and the bioenergetic derangements (5). The changes in renal microcirculation are similar to the profound alteration in the distribution of systemic microvascular blood flow. The renal endothelium is both the victim and the active participant in the pathogenesis of S-AKI (6). The endothelial cells (ECs) are hyperactivated in the presence of proinflammatory cytokines and their structural and functional changes further sustain the progression of sepsis (7). Although aggressive fluid resuscitation and hemodynamic support are used, the mortality of severe septic patients remains high. Therefore, new treatments aiming to reverse endothelial dysfunction and its associated microcirculation abnormalities in sepsis may be promising.

Stem cell-based therapies have received much interest for their great potential to treat inflammation and organ failure-related diseases. In the past few years, many investigations found that perinatal cells, such as human amnion epithelial cells (hAECs), represent a novel class of stem cells. hAECs have trimesodermal lineage differentiation ability under appropriate stimulation *in vitro*; also, hAECs are non-immunogenicity due to the low expression levels of human leukocyte antigens (8). In addition, hAECs have specific advantages on clinical translation, including rich sources, non-invasive operation, easy isolation, huge amount of numbers, genetically stable, and no ethical debates (9). Together, these properties make hAECs hold the potential to ameliorate the burden of critical illness.

Recently, it has emerged that exosomes (EXOs) derived from hAECs appear to exert therapeutic benefits similar to hAECs in preventing organ dysfunction by delivering RNA- and protein-containing cargos to target cells (10–13). In this study, we aim to examine whether administration of hAECs on experimental sepsis by cecal ligation and puncture (CLP) has the therapeutic efficiency and further explore the potential of hAECs and their derived EXOs on inflammatory modulation and EC structural and functional restoration in S-AKI.

## Materials and Methods

### Mice

Male C57BL6/J mice (8–12 weeks) were purchased from SPF (Beijing) Biotechnology Corporation Ltd. [License No. SCXK (Jing) 2019-0010]. All the mice were housed in the specific pathogen-free environment of laboratory animal facility (12 h light/dark cycle and room temperature 25°C) at least 1 week before experiment. Mice were free access to food and water. All the experiments were performed by the Guide for the Care and Use of Laboratory Animals. All the animal experimental procedures were approved by the Laboratory Animal Ethics Committee of Peking University First Hospital (Approval Number: J201901).

### Cecal Ligation and Puncture Model of Sepsis

Cecal ligation and puncture-induced sepsis was performed as previously described (14). Briefly, 8–12 weeks old C57BL/6J male mice were anesthetized with 0.5% pentobarbital sodium (50–60 mg/kg) by intraperitoneal injection. After shaving the abdomen, the mouse was placed in a supine position on an operation pad and sterilized with 75% alcohol. To build sepsis model, a 0.5–1.0 cm abdominal midline incision was cut along the linea alba and the cecum was exteriorized. 75% of the cecum was ligated (distal ligation) with a 4–0 silk suture and penetrated through and through with a 21-gauge needle. One droplet of feces was gently squeezed from penetration holes to ensure patency and then the cecum was relocated into the peritoneal cavity. The abdominal incision was closed in two layers with 5–0 silk sutures and sterilized with iodine. In sham-operated mice, the cecum was only exteriorized as described, without ligation and puncture. 1.0 ml prewarmed saline was injected subcutaneously to resuscitate the mouse. Whole blood sample was collected by cardiac puncture and serum was separated by centrifugation at 2,500 rpm for 10 min at 4°C. Serum creatinine, alanine aminotransferase (ALT), and aspartate aminotransferase (AST) levels were measured by Creatinine Assay Kit, ALT Assay Kit, and AST Assay Kit (all from Jiancheng Bioengineering Institute, Nanjing, China).

### Human Amnion Epithelial Cells Culture and Administration

Human amnion epithelial cells used in this study were provided by Shanghai iCELL Biotechnology Corporation Ltd. (Shanghai, China). The isolation of hAECs had been described in the previous study (15). hAECs were isolated from fresh amniotic membranes from healthy mothers after cesarean deliveries with a written informed consent. The procedure was approved by the Institutional Ethics Committee of the International Peace Maternity and Child Health Hospital, School of Medicine, Shanghai Jiao Tong University (Approved Number: [2014]11). The standard culture medium used was Dulbecco’s Modified Eagle Medium/Nutrient Mixture F-12 (DMEM/F-12) supplemented with 10% fetal bovine serum (FBS), 2 mM L-glutamine, 1% streptomycin-penicillin (all from Gibco, Carlsbad, CA, United States), and 10 ng/ml recombinant human epidermal growth factor (Peprotech). The P1 hAECs were resuspended in vehicle [phosphate-buffered saline (PBS) plus albumin] at a concentration of 1 × 10^7^ cells/ml for administration.

Mice were randomly divided into the three groups: (1) the sham operated group, (2) the CLP + vehicle group, and the (3) the CLP + hAECs group. Vehicle (100 μl) or hAECs (1 × 10^6^ cells per mouse in 100 μl vehicle) were slowly infused *via* tail vein immediately after mice had undergone the CLP operation.

### Mice Status Scoring

The survival rate was assessed every 6–8 h and the observation was terminated at the end of the seventh day. The mice status of 16 h after CLP, which represents the early stage of sepsis, was scored by parameters of mental condition, spontaneous activity, and muscle strength. The scoring system is given in Supplementary Table 1.

### Histology of Organs

Kidney, liver, heart, and lung specimens were fixed in 10% formaldehyde for at least 24 h. After dehydration in a series of ascending concentrations of ethanol, samples were embedded in paraffin and sectioned at 4 μm thickness for staining with H&E. The extent of histopathology damage was scored semiquantitatively by a pathologist who was blind to the experimental groups. Organ damage scoring was based on a 0 to 4 scale and observed by light microscopy (Leica DM2500, Germany) at high power fields (magnification 400 ×). 10–15 randomly selected non-overlapping fields were scored for each mouse and the average for each group was analyzed. Kidney injury was evaluated according to the expansion of Bowman’s capsule (0, no lesion; 1, lesions <10%; 2, lesions ≥10 to 20%; 3, lesions ≥20 to 30%; and 4, lesions ≥30% of the cortex), the vacuolization, and loss of brush border of renal tubular epithelial cells (0, no lesion; 1, lesions <25%; 2, lesions ≥25 to 50%; 3, lesions ≥50 to 75%; and 4, lesions ≥75% of the cortex) (16). Liver injury was evaluated according to sinusoidal congestion, vacuolization of hepatocyte cytoplasm, and parenchymal necrosis (0, no lesion; 1, lesions <25%; 2, lesions ≥25 to 50%; 3, lesions ≥50 to 75%; and 4, lesions ≥75% of the section). Heart injury was evaluated according to myocardial cells edema and vacuolization (0, no lesion; 1, lesions <25%; 2, lesions ≥25 to 50%; 3, lesions ≥50 to 75%; and 4, lesions ≥75% of the section). Lung injury was evaluated according to alveolar congestion and hemorrhage and infiltration of inflammatory cells (0, no lesion; 1, lesions <25%; 2, lesions ≥25 to 50%; 3, lesions ≥50 to 75%; and 4, lesions ≥75% of the section).

### Transmission Electron Microscopy

For transmission electron microscopy (TEM) sample preparation, 1.0 mm^3^ kidney sample was fixed with 2.5% glutaraldehyde at 4°C overnight and washed 3 times in 0.1 M cacodylate buffer, then fixed in 1% osmium tetroxide for 2 h, and dehydrated in 30, 50, 70, 80, 90, and 100% acetone solutions. Samples were cut into 50 nm sections using an ultrathin microtome (Leica EM UC7, Wetzlar, Germany), contrasted with 2% uranyl acetate for 10 min and lead citrate for 5 min, and observed under TEM (JEM-1400, JEOL Ltd., Tokyo, Japan).

### Cytokine and Chemokine Quantification

MILLIPLEX MAP Mouse Cytokine/Chemokine Magnetic Bead Panel—Premixed 32 Plex—Immunology Multiplex Assay (MCYTMAG-70K-PX32, Millipore) was used to quantify the concentration of 32 cytokines and chemokines in mouse plasma: Eotaxin/C-C motif chemokine ligand 11 (CCL11), granulocyte colony stimulating factor (G-CSF), granulocyte-macrophage colony stimulating factor (GM-CSF), interferon-γ (IFN-γ), interleukin-1α (IL-1α), interleukin-1β (IL-1β), interleukin-2 (IL-2), interleukin-3 (IL-3), IL-4, IL-5, IL-6, IL-7, IL-9, IL-10, IL-12 (p40), IL-12 (p70), IL-13, IL-15, IL-17A/cytotoxic T-lymphocyte-associated antigen 8 (CTLA8), inducible protein-10 (IP-10)/C-X-C motif chemokine 10 (CXCL10), Kc/CXCL1, leukemia inhibitory factor (LIF), lipopolysaccharide-induced CXC chemokine (LIX), monocyte chemoattractant protein-1 (MCP-1)/C-C motif chemokine ligand 2 (CCL2), macrophage-stimulating factor (M-CSF), monokine induced by gamma interferon (MIG)/CXCL9, macrophage inflammatory protein 1 (MIP-1α)/CCL3, MIP-1B/CCL4, MIP-2/CXCL2, regulated upon activation normal T cell expressed and presumably secreted (RANTES)/CCL5, tumor necrosis factor-α (TNF-α), and vascular endothelial growth factor A (VEGF-A). Measurements were performed on MAGPIX multiplexing instrument (Luminex Corporation, Austin, TX, United States). Quality control procedures were used to ensure validity.

### Immunohistochemistry Staining

For immunohistochemistry staining, the kidney sections were dewaxed, rehydrated, and antigen retrieved by autoclave in Tris/ethylenediaminetetraacetic acid (EDTA) buffer (pH 9.0). The endogenous peroxidase was blocked by incubation with 3% hydrogen peroxide for 10 min and washing with PBS 3 times. Non-specific binding was blocked with 3% bovine serum albumin (BSA) for 1 h at room temperature. Tissue sections were, respectively, incubated with rabbit anti-vascular cell adhesion molecule-1 (VCAM-1) antibody (1:200, Abcam, ab134047) overnight at 4°C. After washing 3 times with PBS, the tissue sections were incubated with horseradish peroxidase (HRP)-conjugated goat antirabbit secondary antibody (Zhongshan Gloden Bridge Biotechnology, Beijing, China) for 20 min at room temperature, then stained with a 3,3’-diaminobenzidine (DAB) kit (Zhongshan Gloden Bridge Biotechnology, Beijing, China), and visualized under Leica microscope (Germany). Image Pro Plus (IPP) version 6.0 was used to assess the location and intensity of the positive area.

### Isolation and Characterization of Human Amnion Epithelial Cells-Derived Exosomes

The isolation and identification of EXOs had been described in our previous study (15). Briefly, serum-free hAECs culture medium was collected and experienced serial centrifugation at 2,000 *g* for 30 min and 20,000 g (Beckman Coulter, United States) for 30 min at 4°C and 1,50,000 *g* for 70 min at 4°C twice to obtain EXOs. The quality of extracted EXOs was determined by TEM (Zeiss, Oberkochen, Germany) and the expression of EXO markers such as CD63, tumor susceptibility 101 (TSG101), and Alix. Nanoparticle tracking analysis (NTA) was performed to determine the size and concentration of the purified vesicles (Particle Metrix, Meerbusch, Germany) (Supplementary Figure 1). 1 × 10^8^ EXOs resuspended in PBS were injected into the mice intravenously right after CLP operation.

### Human Umbilical Vein Endothelial Cells Cell Culture and Treatment

Human umbilical vein endothelial cells were purchased from iCell Bioscience Incorporation (Shanghai, China) and cultured in complete endothelial cell medium (ECM) (ScienCell, 1001, United States) supplemented with 5% FBS, 1% endothelial cell growth supplement (ECGS), and 1% penicillin/streptomycin at 37°C in a 5% CO_2_ humidified incubator. When grown to 100% confluency in 6 well plates or transwell chambers, human umbilical vein endothelial cells (HUVECs) were starved for 24 h in ECM without serum, followed by stimulation of 1 μg/ml lipopolysaccharide (LPS) (Sigma-Aldrich, L6529) with or without 6.7 × 10^5^/ml EXOs for 24 h.

### Transwell PET Membrane Fluorescence Staining

Polythylene terephthalate (PET) membrane was detached from transwell and washed in PBS 3 times. Membranes were fixed in 4% paraformaldehyde for 20 min at room temperature. HUVECs were permeabilized by 0.1% Triton X-100 in PBS for 5 min, washed with PBS 3 times, and then blocked with 3% BSA in PBS for 1 h at room temperature. The cells were labeled with zonula occludens-1 (ZO-1) primary antibody (Invitrogen, 61-7300, 1:50) overnight at 4°C. After washing, the cells were incubated with donkey antirabbit (Alexa Fluor 488) secondary antibody (1:500 dilution) and rhodamine phalloidin (1:1,000 dilution) for 1 h at room temperature. The membranes were mounted in mounting medium with 4′,6-diamidino-2-phenylindole (DAPI) (ZLI-9557, ZSGB-BIO, China) and coverslipped. Photos were captured by confocal microscope (Zeiss 780, Germany).

### Fluorescein Isothiocyanate-Dextran Assay

A total of 25 mg/ml fluorescein isothiocyanate (FITC)-dextran (4 kDa) (Chondrex, 4013) stock solution was diluted with ECM to 1 mg/ml and was added to the upper chamber of the transwell. After incubation for 2 h at 37°C, medium in the lower chamber was collected and diluted with PBS (1:5) for detection of fluorescence signal at 488 nm (Synergy H1, Gene Company Limited, Hong Kong, China). ECM without FITC-dextran was used as baseline detection.

### Real-Time Reverse Transcription-PCR

Total RNA was extracted from the kidney specimens using TRIzol Reagent following the manufacturer’s instructions (Invitrogen, Carlsbad, CA, United States). RNA concentration was measured by NanoDrop (ND-1000 spectrophotometer, Thermo Fisher Scientific, Wilmington, DE, United States). Complementary DNA (cDNA) was synthesized from 2 μg of total RNA using the FastKing RT Enzyme (KR118; TIANGEN Biotech, Beijing, China) for reverse transcription. Real-time PCR (RT-PCR) reagents were prepared from the SYBR Green PCR Master Mix (FP209; TIANGEN Biotech, Beijing, China) and real-time PCR reactions were performed on an ABI Vii7 System. Results were normalized to glyceraldehyde-3-phosphate dehydrogenase (*Gapdh*) or beta-actin (*ACTB*) and analyzed by the 2^–ΔΔCT^ method (*n* = 4/group). The primer sequences are given in Supplementary Table 2.

### Western Blot

Kidney tissues were lysed on ice in radio immunoprecipitation assay lysis buffer (RIPA) buffer supplemented with protease inhibitors. HUVECs protein was extracted after the cells were treated with LPS or LPS plus EXOs for 24 h. Protein concentrations were determined by the bicinchoninic acid (BCA) method. 30 μg of total denatured protein for each sample was subjected to 4–20% sodium dodecyl sulfate-polyacrylamide gel electrophoresis (SDS-PAGE) gel and transferred to poly 1,1-difluoroethylene (PVDF) membrane. The membranes were blocked with 5% BSA or 5% non-fat dry milk solution at room temperature for 1 h. For immunoblotting, the membranes were incubated with primary antibodies against phospho-p65 (1:1,000, CST, 3033), p65 (1:1,000, CST, 8242), VCAM-1 (1:1,000, Abcam, ab134047), ZO-1 (1:1,000, Affinity, AF5145), and β-actin (1:5,000, Proteintech, 20536-1-AP) overnight at 4°C. Then, it incubated with the secondary antibodies—HRP-conjugated antirabbit immunoglobulin G (IgG) antibody at room temperature for 1 h. The specific bands were detected by enhanced chemiluminescence (ECL) methods and measured by the Image J software (NIH, Bethesda, MD, United States). The levels of β-actin were used as internal standards, respectively.

### Statistical Analysis

Data are expressed as the mean ± SEM. Comparison among groups was made by a one-way ANOVA. The survival rate in each group was presented as the Kaplan–Meier curves and assessed by the log-rank test. The statistical software used were GraphPad Prism version 8.3.0 (San Diego, CA, United States) and Statistical Package for Social Sciences (SPSS) version 24.0 (IBM Corporation, Armonk, NY, United States). *P* < 0.05 was defined as statistically significant.

## Results

### Human Amnion Epithelial Cells Reduced the Mortality and Improved the Clinical Syndrome of Sepsis Mice

**FIGURE 1:**
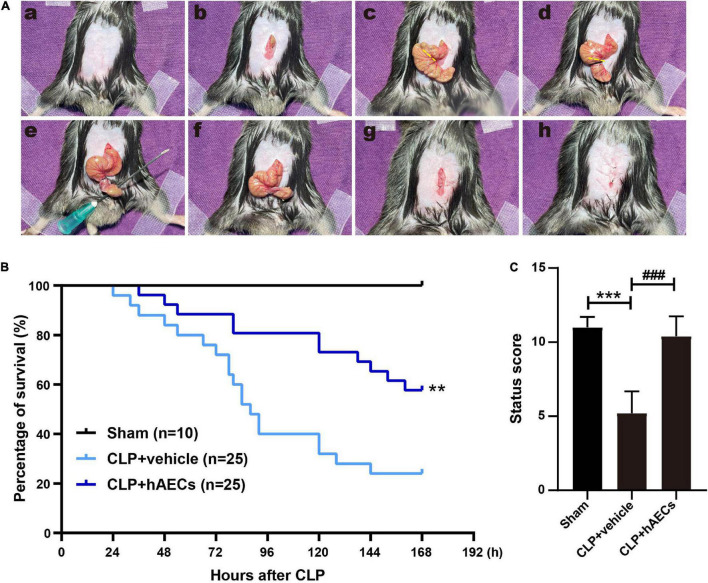
Establishment of septic mouse model and the effects of human amnion epithelial cells (hAECs) on cecal ligation and puncture (CLP) mice. **(A)** Critical steps in the CLP operation in mice. First, the abdominal area was disinfected after shaving (a), the midline skin was incised (b), and the cecum was exposed (c). High-grade sepsis was induced by ligation of 75% of the cecum (d). Then, cecal puncture (through and through) was performed using 21-gauge needle (e). After removing the needle (f), the wound was closed (g) and the abdominal skin was sutured (h). **(B)** Survival rate after induction of sepsis by CLP in mice. **P < 0.01 vs. the CLP + vehicle group. **(C)** Assessment of the clinical signs of mice 16 h after CLP. ***P < 0.001 vs. the sham group, ###*P* < 0.001 vs. the CLP + vehicle group.

To mimic the pathophysiological changes in septic patients, we performed CLP operation in mice (14), which is the most widely used model for experimental sepsis and is currently considered as the gold standard in septic research. High-grade sepsis was induced by ligation of 75% of the cecum (Figure 1A). Within 7 days (168 h) of the observation time, there was 76% mortality in the CLP group treated with vehicle control. However, hAECs administration by mouse tail injection right after CLP operation reduced the 7-day mortality to 42% (Figure 1B).

We constructed a scoring system including aspects of mental state, autonomous motor activity, and myodynamia to evaluate the whole-body status of the septic mice (Supplementary Table 1) after 16 h CLP operation, a time point without any mouse death. The CLP mice received a significantly lower status score with symptoms of low body temperature, listlessness, hunched back, wobbly walk, muscle weakness, and decreased autonomic reflex. hAECs administration reversed those symptoms and improved the body status to be comparable to the sham group (Figure 1C).

### Effect of Human Amnion Epithelial Cells on Sepsis-Induced Injury of the Kidney and Other Organs

**FIGURE 2:**
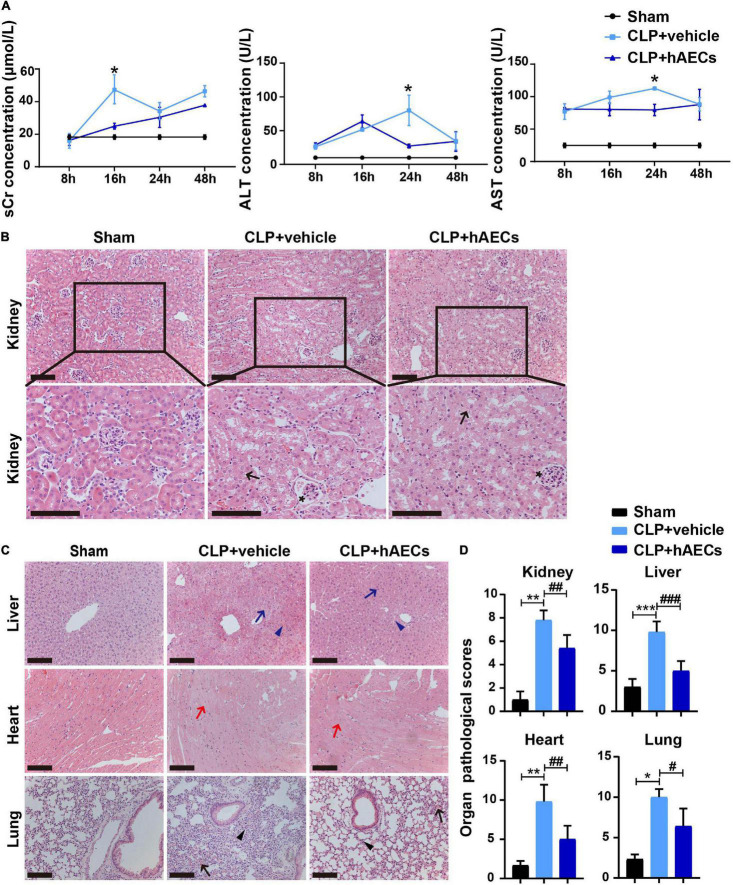
hAECs ameliorated multiple organ damage of CLP mice. **(A)** Serum creatinine, alanine aminotransferase, and aspartate aminotransferase concentrations at different times in mice with CLP operation followed by vehicle (*n* = 5) or hAECs (*n* = 5) injection. *P < 0.05 vs. the CLP + vehicle group. **(B)** Renal pathology of septic mice at 16 h after CLP or CLP with hAECs treatment. Representative micrographs from each group are shown. The arrows indicate tubular epithelial vacuolization and the loss of brush border and the asterisks indicate Bowman’s capsule expansion (H&E staining; scale bar = 50 μm). The lesions in the kidney were alleviated by the hAECs treatment. **(C)** Pathology of liver, heart, and lung of septic mice at 16 h after CLP or CLP with hAECs treatment. Representative micrographs from each group are shown. The blue arrows indicate vacuolization of hepatocyte cytoplasm and the blue arrowheads indicate sinusoidal congestion of liver tissues. The red arrows indicate edema and vacuolization of myocardial cells of heart tissues. The black arrows indicate alveolar congestion and hemorrhage. The black arrowheads indicate infiltration or aggregation of neutrophils in airspaces of lung tissues (H&E staining; scale bar = 50 μm). The lesions in the liver, heart, and lung were alleviated by the hAECs treatment. **(D)** Organ pathological scores representing the degree of lesion damage at 16 h after CLP. *P < 0.05, **P < 0.01, ***P < 0.001 vs. the sham group; #*P* < 0.05, ##*P* < 0.01, ###*P* < 0.001 vs. the CLP + vehicle group.

The severity of CLP-induced organ injury was further determined by organ function indicators and by histological evaluation. Serum creatinine (sCr), alanine aminotransferase (ALT), and aspartate aminotransferase (AST) levels were measured to reflect the functional changes of the kidney and the liver. As shown in Figure 2A, the level of sCr was similar in each group 8 h after CLP surgery. However, 16 h after surgery, sCr level was increased significantly and an obvious decrease of sCr was observed in the hAECs treatment group. Besides, ALT and AST were both upregulated in the CLP group and downregulated after the treatment of hAECs in a time-dependent manner (Figure 2A).

Results from H&E-stained kidney tissue slides showed that glomerulus was normal and the proximal tubules were tightly packed in the sham group. However, in the CLP group, the proximal tubules showed tubular epithelial vacuolization and the loss of brush border. Bowman’s capsule expansion of glomerulus was also observed. hAECs treatment alleviated these histological changes as assessed with the renal pathological score (Figures 2B,D). Histological injury of the liver, the lung, and the heart were also evaluated on the H&E-stained tissue slides. Treatment with hAECs could ameliorate organ histological scores when compared to septic mice (Figures 2C,D).

### Effect of Human Amnion Epithelial Cells on Sepsis-Induced Systemic Inflammatory Response and Endothelial Dysfunction

**FIGURE 3:**
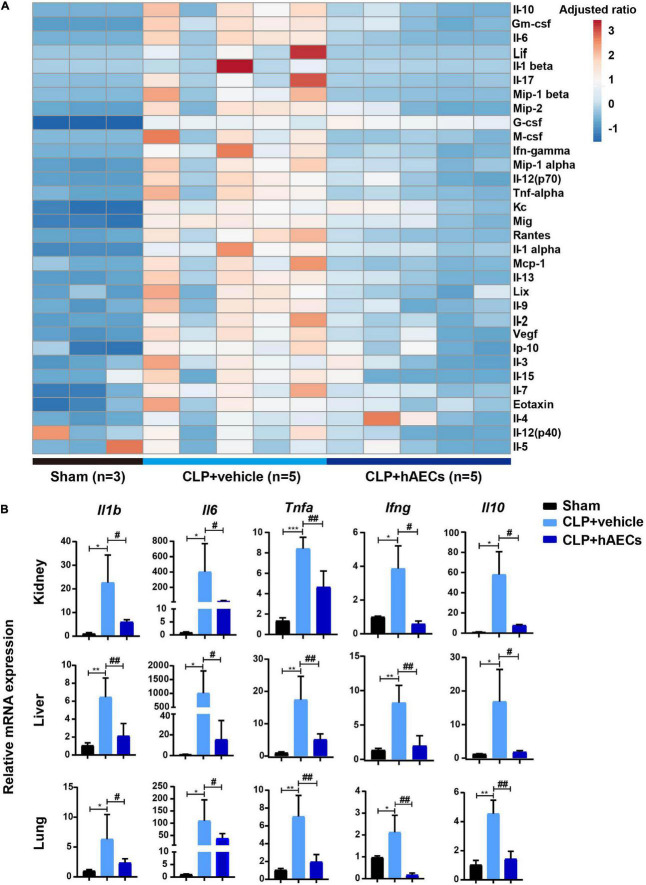
hAECs suppress the levels of inflammatory mediators in septic mice. **(A)** Heat map shown the changes of the protein levels of inflammatory mediators in the plasma of septic mice. **(B)** The messenger RNA (mRNA) expression of inflammatory mediators in kidney, liver, and lung of septic mice 16 h after CLP with hAECs or vehicle administration. *P < 0.05, **P < 0.01, ***P < 0.001 vs. the sham group; #*P* < 0.05, ##*P* < 0.01 vs. the CLP + vehicle group.

**FIGURE 4:**
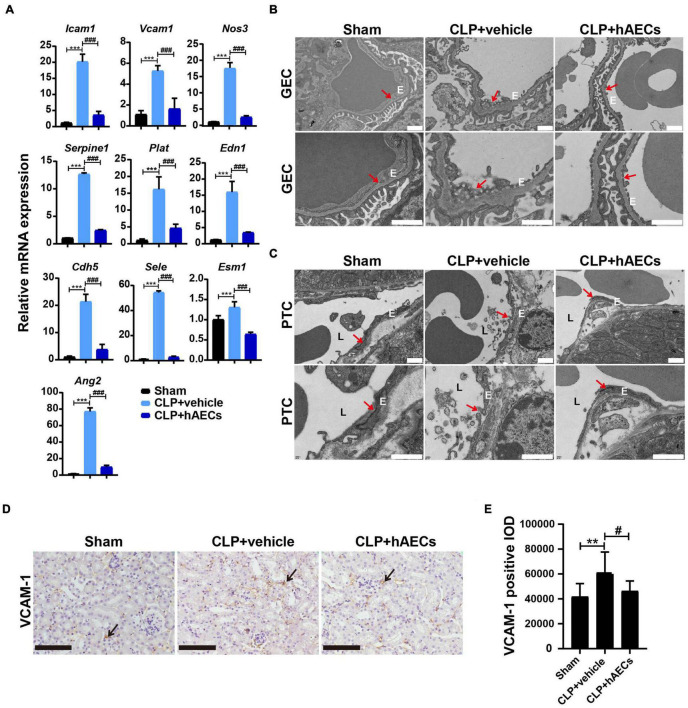
hAECs protected the endothelial cell structure and function from sepsis-associated inflammatory injury. **(A)** The mRNA levels of indicators associated with endothelial cell adhesion and function of septic mice kidney at 16 h after CLP (*n* = 3). ***P < 0.001 vs. the sham group; ###*P* < 0.001 vs. the CLP + vehicle group. **(B)** Electron microscope images shown glomerular endothelial cell (GEC) fenestrae in the CLP mice at 16 h after CLP. The arrows indicate fenestrae of GECs. Scale bar = 1 μm. E: endothelial cell. **(C)** Electron microscope images shown tight junction disruption and the damage of the peritubular capillary endothelial layer in the CLP mice at 16 h after CLP. The arrows indicate tight junctions between two adjacent endothelial cells of the peritubular capillary (PTC). Scale bar = 1 μm. E: endothelial cell. L: peritubular capillary lumen. **(D)** Immunohistochemistry staining of vascular cell adhesion molecule-1 (VCAM-1) in kidney paraffin sections from the indicated groups at 16 h after CLP. Scale bar = 50 μm. Arrows indicate the positive staining. **(E)** Statistical comparison of integrated optical density (IOD) of VCAM-1 positive staining in the indicated groups. **P < 0.01 vs. the sham group; #*P* < 0.05 vs. the CLP + vehicle group.

Inflammatory cytokines in the blood were detected by multiplex assays for protein quantitation. The results in Figure 3A displayed that CLP surgery markedly increased the levels of proinflammatory mediators such as GM-CSF, IL-6, IL-17, MIP-1β, M-CSF, IFN-γ, TNF-α, and MCP-1 as well as the levels of anti-inflammatory mediators such as IL-10 and IL-13, while these changes were significantly reversed by the introduction of hAECs. Additionally, the results in Figure 3B showed that the messenger RNA (mRNA) levels of *Il1b*, *Il6*, *Tnfa*, *Ifng* were higher in the kidney, liver, and lung in the CLP group than those in the control group, hinting the increased systemic inflammatory response after CLP surgery, while hAECs treatment significantly reversed the increased expression caused by CLP surgery. The mRNA levels of markers for endothelial hyperactivation by inflammatory response such as intercellular cell adhesion molecule-1 (*Icam-1*), *Vcam-1*, selectin E (*Sele*), and cadherin 5 (*Cdh5*) and the soluble factors that stimulate endothelial dysfunction including *Esm1*, *Edn1*, *Serpine1*, tissue type plasminogenactivator (*Plat*), angiopoietin-2 (*Ang2*), and nitric oxide synthase 3 (*Nos3*) were markedly increased in the kidney after CLP surgery, while hAECs treatment decreased the expression caused by CLP surgery (Figure 4A). The above results suggested that hAECs could effectively alleviated sepsis-induced systematic inflammation and endothelial dysfunction.

Transmission electron microscopy was used for structural analysis of the endothelial layer of the glomeruli and the peritubular capillaries (Figures 4B,C). The glomerular EC (GEC) fenestrae were destroyed in the CLP mouse kidney, while hAECs treatment restored the normal structure of GEC fenestrae (Figure 4B). The ECs of the peritubular capillaries were closely attached to the basal lamina and the tight junction between two adjacent ECs was clearly observed in the kidney of sham operated mouse. In the CLP mouse kidney, the ECs exhibited irregular shape and detachment of endothelial layers from the basal lamina surface was typically seen. The tight junction was disappeared between the two adjacent ECs. In the group of hAECs treatment after CLP surgery, the tight junction was more frequently detected between the ECs (Figure 4C). Through immunohistochemistry staining, an increase in the VCAM-1 expression was detected in the CLP-operated kidney, which was ameliorated by hAECs treatment, indicating that hAECs could prevent ECs hyperactivation (Figures 4D,E).

### Human Amnion Epithelial Cells-Derived Exosomes Exhibited Similar Renal Protective Effects as Human Amnion Epithelial Cells

**FIGURE 5:**
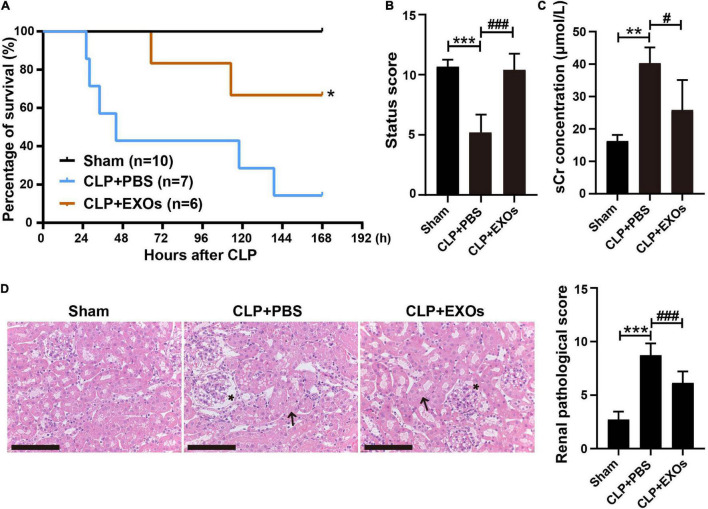
hAECs-derived exosomes (EXOs) reduced the mortality of septic mice and ameliorated kidney damage. **(A)** EXOs reduced the mortality of septic mice. *P < 0.05 vs. the CLP + PBS group. **(B)** Assessment of the clinical signs of mice at 16 h after CLP. ***P < 0.001 vs. the sham group; ###*P* < 0.001 vs. the CLP + phosphate-buffered saline (PBS) group. **(C)** Serum creatinine concentration in mice 16 h after CLP with PBS (*n* = 5) or EXOs (*n* = 5) injection. **P < 0.01 vs. the sham group; #*P* < 0.05 vs. the CLP + PBS group. **(D)** Renal pathology of septic mice kidney at 16 h after CLP or CLP with EXOs treatment and renal pathological scoring. Representative micrographs from each group are shown. The arrows indicate tubular epithelial vacuolization and the loss of brush border and the asterisks indicate Bowman’s capsule expansion (H&E staining; scale bar = 50 μm). The lesions in the kidney were alleviated by the EXOs treatment. ***P < 0.001 vs. the sham group; ###*P* < 0.001 vs. the CLP + PBS group.

Increasing evidences have shown that stem cells typically exert their therapeutic effects through paracrine secretion of extracellular vesicles such as EXOs, rather than directly repopulating into the damaged tissues. We isolated EXOs from hAEC conditioned medium and tested their effects on S-AKI. As shown in Figure 5A, injection of EXOs reduced the 7-day (168 h) mortality of CLP mice from 86 to 33% and improved the whole-body condition of CLP mice (Figures 5A,B), which was comparable to the effects of hAECs (Figures 1B,C). sCr levels were significantly decreased in the EXOs treatment group 16 h after CLP surgery, indicating a recovery of kidney function (Figure 5C). Renal pathological score was also reversed after EXOs administration, suggesting the alleviation of the renal histological injury (Figure 5D). The pathological damages of the liver and lung in the CLP mice were also mitigated by EXOs treatment (Supplementary Figure 2).

### Exosomes Stabilized Endothelial Monolayer Integrity and Suppressed Nuclear Factor Kappa B Pathway Activation

**FIGURE 6:**
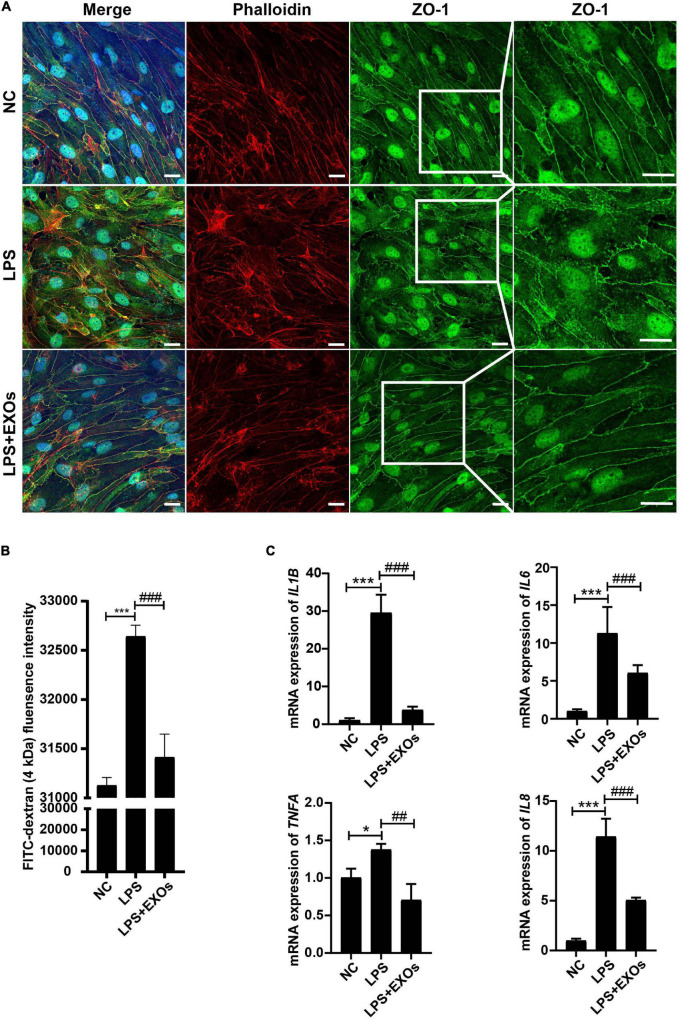
EXOs had beneficial effects on endothelial integrity. **(A)** Confocal images of human umbilical vein endothelial cells (HUVECs) grown on transwells and treated with lipopolysaccharide (LPS) or LPS plus EXOs. Scale bar = 20 μm. **(B)** Fluorescein isothiocyanate (FITC)-dextran permeability assay of HUVECs on transwells in the indicated groups (*n* = 3). ***P < 0.001 vs. the normal control (NC) group; ###*P* < 0.001 vs. the LPS group. **(C)** The mRNA expression of inflammatory mediators in HUVECs 24 h after LPS stimulation and EXOs treatment. *P < 0.05, ***P < 0.001 vs. the NC group; ##*P* < 0.01, ###*P* < 0.001 vs. the LPS group.

To investigate the role of EXOs on the endothelial monolayer integrity after endothelial cell injury, HUVECs grown on transwells were stimulated with 1 μg/ml LPS or LPS plus EXOs for 24 h, after which the cells were stained for F-actin (phalloidin) and ZO-1. As shown in Figure 6A, strong peripheral F-actin and lateral cell surface staining of ZO-1 were observed in the normal control (NC) group. LPS treatment induced the loss of the peripheral F-actin staining and the distortion of ZO-1 linear border pattern. EXOs treatment reserved the strong cell border signal of F-actin and ZO-1. Furthermore, endothelial monolayer permeability was determined by FITC-dextran transwell assay. FITC-dextran fluorescence was detected in the lower chamber 24 h after LPS stimulation. Compared with that in NC samples, LPS treatment induced a significant increase in the leakage of 4-kDa FITC-dextran from the endothelial monolayer, while EXOs reduced FITC-dextran leakage, indicating EXOs could conserve the integrity of endothelial monolayer (Figure 6B). As shown in Figure 6C, EXOs also decreased the mRNA expression of LPS-induced proinflammatory cytokines such as *IL1B*, *TNFA*, *IL6*, and *IL8*. Together, these results showed the protective role of EXOs on endothelial permeability in HUVEC monolayers *in vitro*.

**FIGURE 7:**
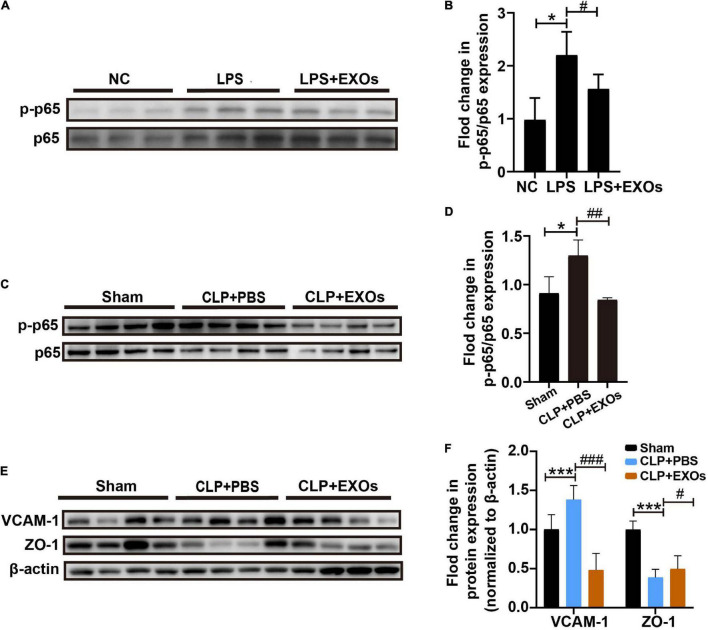
EXOs suppressed nuclear factor kappa B (NF-κB) pathway activation. **(A)** Western blot of phosphor-p65 (p-p65) protein level in cultured HUVECs treated with LPS or LPS plus EXOs. **(B)** Semiquantification of Western blot from HUVECs (*n* = 3). *P < 0.05 vs. the NC group; #*P* < 0.05 vs. the LPS group. **(C)** p-p65 and p65 expression in mice kidneys 16 h after CLP with PBS or EXOs injection. **(D)** Semiquantification of Western blots from kidney samples (*n* = 4). *P < 0.05 vs. the sham group; ##*P* < 0.01 vs. the CLP + PBS group. **(E)** VCAM-1 and zonula occludens-1 (ZO-1) expression in mice kidneys 16 h after CLP with PBS or EXOs injection. **(F)** Semiquantification of Western blots from kidney samples (*n* = 4). ***P < 0.01 vs. the sham group; #*P* < 0.05, ###*P* < 0.001 vs. the CLP + PBS group.

To determine whether EXOs prevent the endothelial dysfunction by LPS stimulation through the regulation of inflammatory response signaling pathways, we detected Nuclear Factor Kappa B (NF-κB) signaling activation. The levels of p65 phosphorylation were checked by Western blot. The results showed that LPS treatment remarkably increased the levels of p-p65 (Figures 7A,B) compare to those under NC conditions and EXOs suppressed the NF-κB pathway activation. In consistent with the *in vitro* results, EXOs treatment in CLP mice also decreased the p65 phosphorylation (Figures 7C,D) and corrected endothelial hyperactivation as indicated by changes in VCAM-1 and ZO-1 signals (Figures 7E,F).

## Discussion

According to the data from the Global Burden of Disease, Injuries, and Risk Factors Study (GBD) 2017, an estimated 48.9 million incident cases of sepsis were recorded worldwide and 11.0 million sepsis-related deaths were reported, representing 19.7% of all the global deaths (17). Although any organ system can be affected in sepsis, in clinical practice, six organ systems are usually evaluated including the cardiovascular, respiratory, renal, neurological, hematological, and hepatic systems (18). The current treatment regimen for patients with sepsis consists of rapid control and removal of the source of infection, warranty of hemodynamic stabilization, and organ supporting (19, 20). Despite advances in supportive care, sepsis is still associated with very high mortality (40–60%) and is the leading cause of death in non-coronary intensive care units (3).

Sepsis and AKI are often present simultaneously. S-AKI is the most common AKI syndrome in intensive care units (ICUs) and accounts for approximately half of AKI entity (21). Despite extensive research and multiple randomized clinical trials, only few new therapies were found to be relatively beneficial (22, 23) to sepsis. In the recent years, a variety of stem cells have been reported to have therapeutic effects on experimental septic animal models such as mesenchymal stem cells (MSCs) of different sources (derived from bone marrow, adipose tissue, umbilical cord, or amniotic fluid), induced pluripotent stem cell (iPSCs), and endothelial progenitor cells (EPCs) (24–28), yet the tumorgenicity of those stem cells is always a major concern. hAECs, as a new source of perinatal stem cells, can be isolated from the innermost layer in term placenta. hAECs possess embryonic stem cell markers, but lack telomerase activity, suggesting no risk of tumor formation (29). The role of hAECs is increasingly recognized in many disease settings over the last years, for example, wound healing (30), multiple sclerosis (31, 32), lung injury (11, 33), liver fibrosis (34, 35), ischemia-reperfusion-induced AKI (IRI-AKI) (15), myocardial infarction (36), and premature ovarian failure (37). Previously, this study has demonstrated that hAECs could improve survival and ameliorate renal injury in mice with IRI-AKI through immunomodulation, antiapoptosis, and prevention of peritubular capillary loss (15). In this study, we found that hAECs could decrease the mortality rate of CLP mice, ameliorate septic injury in the kidney and other organs, and improve kidney and liver function. Recently, special attention has been paid to the EXOs derived from stem cells, which can act as a shuttle from parent cells to target cells and have the same biological activity as the parent cells themselves (38–40). We have observed that EXOs from hAECs also increased the survival rate of CLP mice, improved renal function and relieved renal damage.

When compared with AKI of non-septic origin, S-AKI is characterized by a distinct pathophysiology at least in the first 48 h with a concomitant pro- and anti-inflammatory state and lack of histological changes of the kidney (41). Inflammation, microcirculatory dysfunction, and metabolic reprogramming have been proposed as new mechanisms to explain the dissociation between structural and functional changes. Microcirculatory dysfunction is associated with the heterogeneous changes in regional kidney perfusion and oxygenation (42, 43) and may be partially due to endothelial activation (44). Inflammatory cytokines induce endothelial cell hyperactivation with increased secretion of adhesion molecules and promotion of the rolling and adhesion of leukocytes and platelets, leading to an increased risk of thrombi formation and altered flow continuity. Furthermore, endothelial activation is associated with an increased vascular permeability and leakage, causing interstitial edema and increasing hypoxia to the tubular epithelial cells. Therefore, therapeutic strategies targeting the immunomodulation and microcirculation correction may be necessary for better treatment outcomes. Mechanistically, we observed that hAECs and EXOs suppressed systemic inflammation and maintained the renal endothelial integrity in septic mice. More precisely, EXOs suppressed LPS-induced proinflammatory NF-κB pathway activation and maintained endothelial cell adhesion junction *in vitro*. *In vivo* EXOs also protected against sepsis-induced NF-κB pathway activation and endothelial hyperactivation.

Compared to stem cell-based therapy, exosome therapy offers more advantages, including no immune response, no potential tumorigenicity, stable and suitable for long-term storage, and delivering stronger signaling in intercellular communication, etc. (45, 46). Therefore, standardized the culture condition and isolation protocol, identification of the beneficial contents in EXOs from hAECs, and confirming the potential effects of EXOs in the late stage of sepsis will be the future working directions for hAECs-derived EXOs translational study.

## Data Availability Statement

The original contributions presented in the study are included in the article/Supplementary Material, further inquiries can be directed to the corresponding authors.

## Ethics Statement

The studies involving human participants were reviewed and approved by the Institutional Ethics Committee of the International Peace Maternity and Child Health Hospital, School of Medicine, Shanghai Jiao Tong University (Approved Number: [2014]11). The patients/participants provided their written informed consent to participate in this study. The animal study was reviewed and approved by Laboratory Animal Ethics Committee of Peking University First Hospital (Approval Number: J201901).

## Author Contributions

DC and YC performed the experiments, collected, analyzed, and interpreted data. YC drafted the manuscript. HW completed the semiquantitation of organ injury. WY, XZ, DX, NL, and MX helped assemble the data. CX, SW, and GL helped revise the manuscript. SL and LY conceived and supervised the study, interpreted the data, and revised the manuscript. All the authors have read and approved the final manuscript.

## Conflict of Interest

LY, SL, YC, and CX have patents pending for the use of hAECs in repair of the injured kidney after AKI. The hAECs used in this study were provided by Shanghai iCELL Biotechnology Corporation Ltd. (Shanghai, China). The funder was not involved in the study design, collection, analysis, interpretation of data, the writing of this article or the decision to submit it for publication. The remaining authors declare that this study was conducted in the absence of any commercial or financial relationships that could be construed as the potential conflicts of interest.

## Publisher’s Note

All claims expressed in this article are solely those of the authors and do not necessarily represent those of their affiliated organizations, or those of the publisher, the editors and the reviewers. Any product that may be evaluated in this article, or claim that may be made by its manufacturer, is not guaranteed or endorsed by the publisher.
