# Supplementary figures and images for: Human Amnion Epithelial Cells and Their Derived Exosomes Alleviate Sepsis-Associated Acute Kidney Injury via Mitigating Endothelial Dysfunction
Source: Front Med (Lausanne). 2022 Mar 24;9:829606. doi: 10.3389/fmed.2022.829606 (PMC8989462; doi:10.3389/fmed.2022.829606)

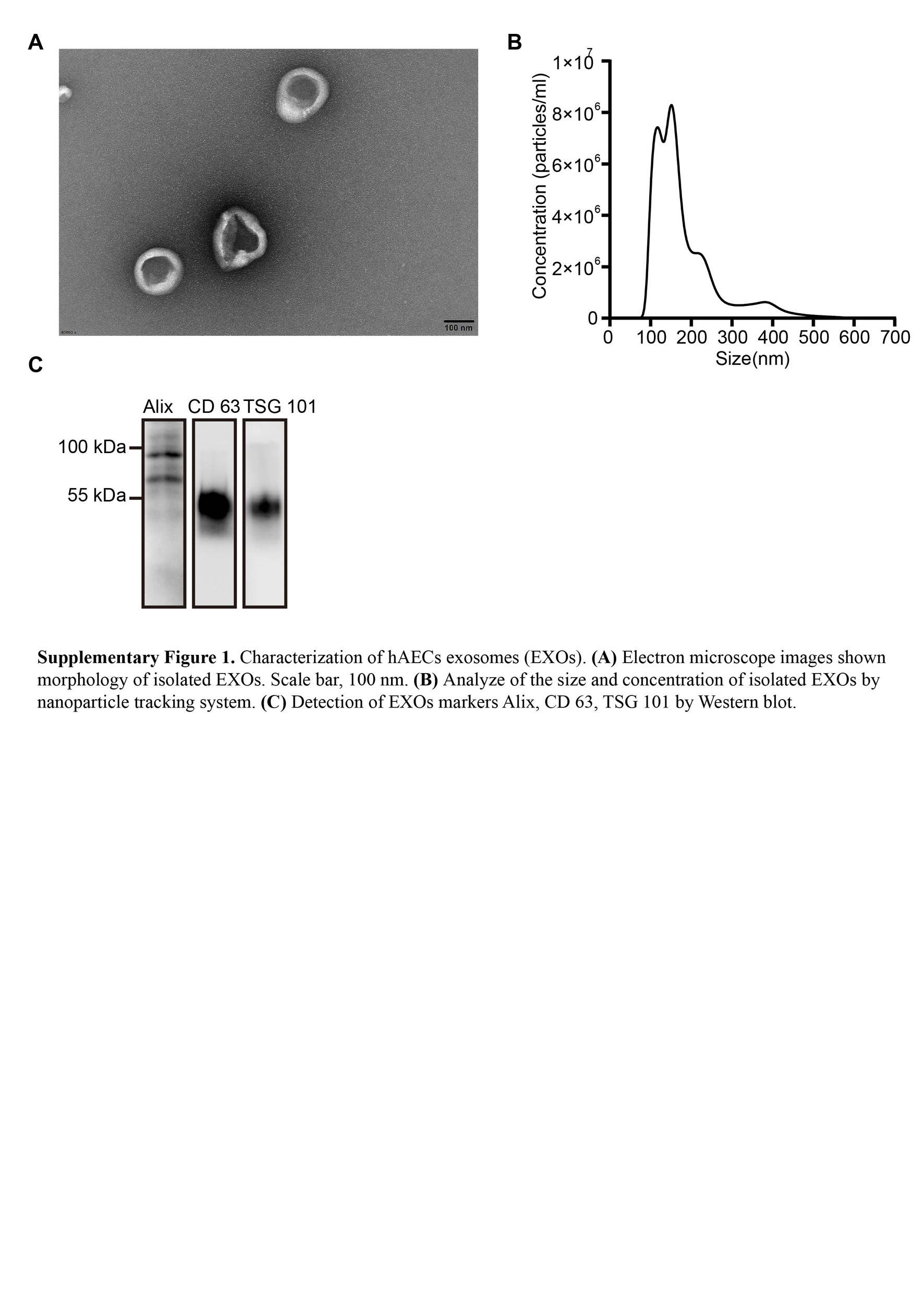

Supplement: Supplementary file 1 [file Image_1.JPEG]

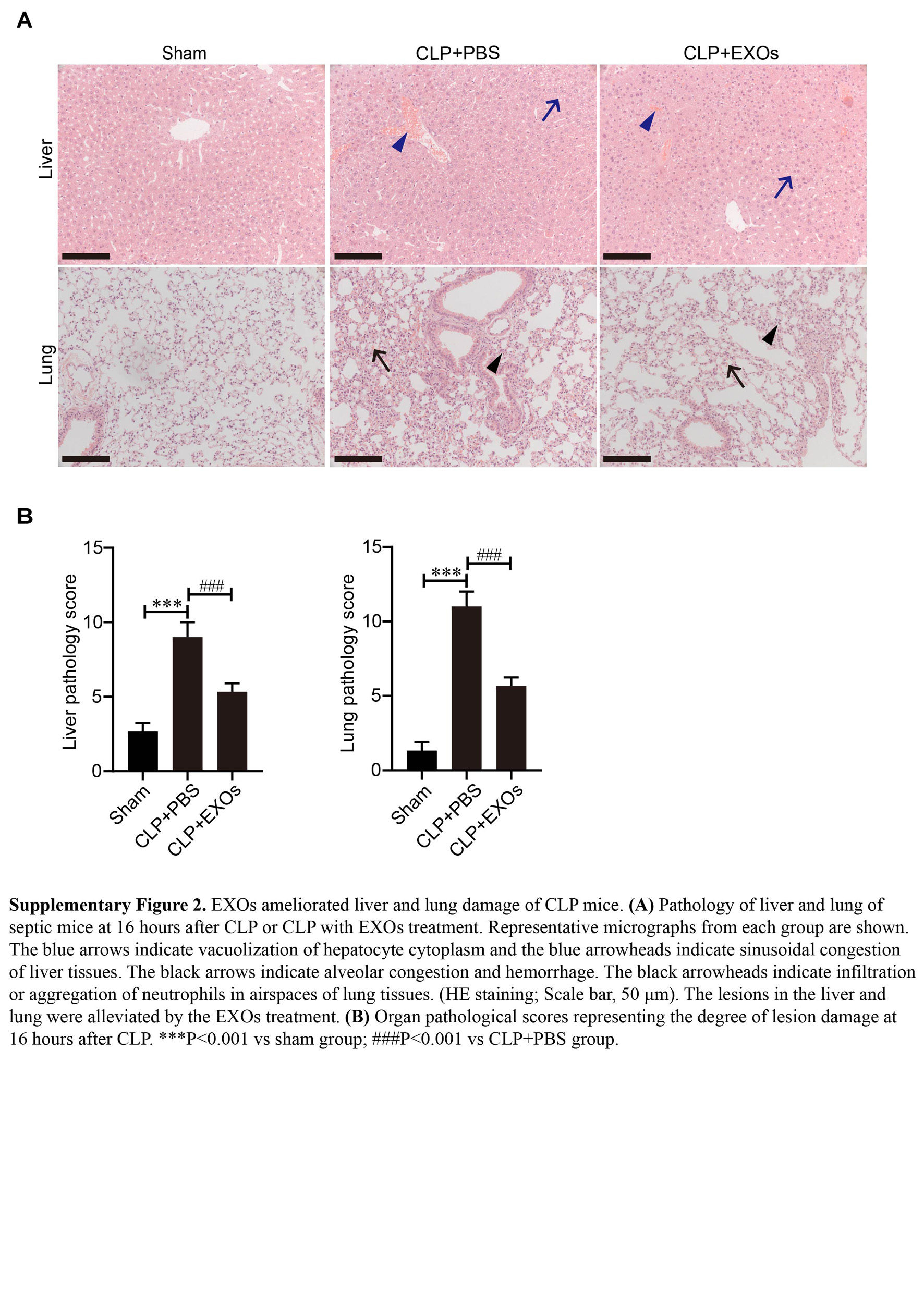

Supplement: Supplementary file 2 [file Image_2.JPEG]
