# Supplementary material for: Human Amnion Epithelial Cells and Their Derived Exosomes Alleviate Sepsis-Associated Acute Kidney Injury via Mitigating Endothelial Dysfunction
Source: Front Med (Lausanne). 2022 Mar 24;9:829606. doi: 10.3389/fmed.2022.829606 (PMC8989462; doi:10.3389/fmed.2022.829606)
Supplement: Supplementary file 3 [file Data_Sheet_1.pdf]

Supplementary Table 1. Mice status scoring

Key	Score
**Mental state**	
General weakness, malaise, piloerection, avoiding conditioned reflex	
Mice have all of the above conditions	0
Mice have three of the above conditions	1
Mice have two the of above conditions	2
Mice have one of the above conditions	3
Mice have none of the above conditions	4
**Autonomous motor activity.**	
Under the condition of knocking the cage	
Mice have no movement	0
Mice have a few intermittent movements	1
Mice have some intermittent movements	2
Mice have consistent movements	3
Mice move autonomously	4
**Myodynamia**	
Put mice on the metal cage and pull the tail	
Mice have no grip and are easy to move	0
Mice have the action of grip, but are easy to move	1
Mice have the action of grip and have a sense of moving forward but can’t against pulling force	2
Mice have the action of grip and move forward but can’t against the force	3
Mice can grip and move forward actively	4

Supplementary Table 2 Primer list

Gene name		Primers
Il1b		
	F’	GAAATGCCACCTTTTGACAGTG
	R’	TGGATGCTCTCATCAGGACAG
Il6		
	F’	CTGCAAGAGACTTCCATCCAG
	R’	AGTGGTATAGACAGGTCTGTTGG
Il10		
	F’	AGCCTTATCGGAAATGATCCAGT
	R’	GGCCTTGTAGACACCTTGGT
Tnfa		
	F’	CCTGTAGCCCACGTCGTAG
	R’	GGGAGTAGACAAGGTACAACCC
Ifng		
	F’	GCCACGGCACAGTCATTGA
	R’	TGCTGATGGCCTGATTGTCTT
Icam1		
	F’	TCCGCTACCATCACCGTGTAT
	R’	TAGCCAGCACCGTGAATGTG
Vcam1		
	F’	TTGGGAGCCTCAACGGTACT
	R’	GCAATCGTTTTGTATTCAGGGGA
Sele		
	F’	ATGAAGCCAGTGCATACTGTC
	R’	CGGTGAATGTTTCAGATTGGAGT
Cdh5		
	F’	CCACTGCTTTGGGAGCCTT
	R’	GGCAGGTAGCATGTTGGGG
Esm1		
	F’	CTGGAGCGCCAAATATGCG
	R’	TGAGACTGTACGGTAGCAGGT
Edn1		
	F’	TTTCCCGTGATCTTCTCTCTGC
	R’	CTGAGTTCGGCTCCCAAGAC
Serpine1		
	F’	TCTGGGAAAGGGTTCACTTTACC
	R’	GACACGCCATAGGGAGAGAAG
Plat		
	F’	TGACCAGGGAATACATGGGAG
	R’	GTCTGCGTTGGCTCATCTCTG
Ang2		
	F’	AGAATAAGCAAGTCTCGCTTCC
	R’	TGAACCCTTTAGAGGCTCGGT

Nos3		
	F'	TCAGCCATCACAGTGTTCCC
	R'	ATAGCCCGCATAGCGTATCAG
Gapdh		
	F'	AGGTCGGTGTGAACGGATTTG
	R'	GGGGTCGTTGATGGCAACA
IL1B		
	F'	ATGATGGCTTATTACAGTGGCAA
	R'	GTCGGAGATTCGTAGCTGGA
IL6		
	F'	AGGGCTCTTCGGCAAATGTA
	R'	GAAGGAATGCCCATTAACAACAA
IL8		
	F'	TTTTGCCAAGGAGTGCTAAAGA
	R'	AACCCTCTGCACCCAGTTTTC
TNFA		
	F'	TGTGTGGCTGCAGGAAGAAC
	R'	GCAATTGAAGCACTGGAAAAGG
ACTB		
	F'	GGCATGGGTCAGAAGGATTCC
	R'	ATGTCACGCACGATTTCCCGC
